# Combination of four bacterial strains isolated from *Yamahai‐shubo* in traditional Japanese sake brewing

**DOI:** 10.1002/fsn3.3280

**Published:** 2023-03-02

**Authors:** Hisashi Fujiwara, Kunihiko Watanabe, Yoshinori Wakai

**Affiliations:** ^1^ Kizakura Co., Ltd Kyoto Japan; ^2^ Division of Applied Life Sciences, Graduate School of Life and Environmental Sciences Kyoto Prefectural University Kyoto Japan

**Keywords:** diacetyl, lactic acid bacteria, sake brewing, *Yamahai‐shikomi* sake, *Yamahai‐shubo*

## Abstract

This study investigated the interactions of four bacteria strains isolated from *Yamahai*‐*shubo*, the source of yeast used to produce a Japanese traditional rice wine, *Yamahai‐shikomi* sake. The bacterial strains were nitrate‐reducing *Pseudomonas* sp. 61‐02, *Leuconostoc mesenteroides* LM‐1, *Lactiplantibacillus plantarum* LP‐2, and *Latilactobacillus sakei* LS‐4. We examined fermentation factors for *Yamahai‐shubo* and *Yamahai‐shikomi* sake samples to compare the suitability of their bacterial combination (16 variations). As a result of principal component analysis, we found that two major groups were formed; one containing strain LP‐2 and the other containing strain LS‐4, and that strains LP‐2 and LS‐4 were important in the *Yamahai‐shikomi* sake in the presence of strains 61‐02 and LM‐1. Then, we investigated the effects of strains LP‐2 and LS‐4 on the concentration of organic acids (pyruvic acid, citric acid, succinic acid, malic acid, and lactic acid) in *Yamahai‐shikomi* sake. Only in lactic acid, a tendency to decrease with a smaller proportion of LS‐4 strains in *Yamahai‐shubo* was observed. Subsequently, their effect on the concentration of diacetyl, crucial for aroma, was investigated between the LP‐2 and LS‐4 strains. The sample prepared in the absence of strain LS‐4 exhibited the lowest concentration of diacetyl. This result was supported by the statistical analysis for the sensory scores performed for aroma of each *Yamahai‐shikomi* sake sample. In conclusion, strain LP‐2 plays a more significant role in improving *Yamahai‐shikomi* sake quality with strains LM‐1 and 61‐02 rather than strain LS‐4 in *Yamahai‐shubo* preparation and *Yamahai‐shikomi* sake brewing.

## Graphical abstract

This study investigated the interactions of four bacteria strains (nitrate‐reducing Pseudomonas sp. 61‐02, *Leuconostoc mesenteroides* LM‐1, *Lactiplantibacillus plantarum* LP‐2, and *Latilactobacillus sakei* LS‐4) isolated from *Yamahai‐shubo*. We examined fermentation factors for *Yamahai‐shubo* and *Yamahai‐shikomi* sake samples to compare the suitability of their bacterial combination (16 variations). In conclusion, strain LP‐2 plays a more significant role in improving *Yamahai‐shikomi* sake quality with strains LM‐1 and 61‐02 rather than strain LS‐4 in *Yamahai‐shubo* preparation and *Yamahai‐shikomi* sake brewing.

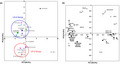

## 1 INTRODUCTION

Japanese sake, a traditional rice wine, is produced by a multistep fermentation process mainly using well‐studied strains of *Saccharomyces cerevisiae* with *Aspergillus oryzae* derived from *koji* (Japan Sake and Shochu Makers Association, 2011). The recent method for brewing sake employs a current starter, a so‐called *Sokujo*‐*shubo* that includes those *S. cerevisiae* strains, and is characterized by a shorter brewing period, although significant amounts of lactic acid are added to prevent contamination by other microorganisms. In contrast, the method of *Yamahai‐shikomi* is one of the traditional methods of Japanese sake brewing (Tatsukami et al., 2018). It employs a traditional *Yamahai‐shubo* that includes various and unidentified species of microorganisms (yeasts and bacteria) that are derived from each brewery. It is known to need more time than *Sokujo‐shubo* because lactic acid is derived from lactobacilli included *Yamahai‐shubo*, and it increases the risk of putrefaction of nonpreferable microorganisms, causing an off‐flavor (Suzuki et al., 2008). On the other hand, it is most likely that the traditional method differentiates the quality of sake by the complex aromas and tastes derived from the microorganisms (Akaike et al., 2020); therefore, the produced sake often improves the original palatability due to the involvement of unidentified microbial habitat(s) in breweries. However, there are uncertain microbial diversities containing various species of bacteria and yeasts in *Yamahai‐shubo*, and the diversities are dependent on the derived sources of breweries (Koyanagi et al., 2016).

The preparation of *Yamahai‐shubo* is usually initiated in a lower temperature environment below 10°C (Obayashi & Kitahara, 1959); as a result, nitrate‐reducing bacteria proceed to grow and convert trace, but significant amounts of nitrate derived from water (groundwater) into nitrite to prevent the growth of nitrite‐sensitive microorganisms. In this early stage of the preparation of *Yamahai‐shubo*, and the condition for bacterial cell growth is insufficient in the presence of nitrate‐reducing bacteria. Thereafter, coccoid‐form lactic acid bacteria (cocci‐LAB), e.g., *Leuconostoc mesenteroides*, gradually and then preferentially grow, producing only a small amount of lactic acid. Subsequently, bacillus‐form lactic acid bacteria (bacilli‐LAB), e.g., *Latilactobacillus sakei* (Ashizawa & Saito, 1966a; Koyanagi et al., 2016), increasingly become predominant, producing a larger amount of lactic acid, which serves to decrease the pH of the medium. After the microbial transition, a well‐studied strain of *S. cerevisiae* is added between 10 and 15 days for the preparation of *Yamahai‐shubo*, while concomitant bacterial species become crucial for the characteristic brewing of *Yamahai‐shikomi* sake. Therefore, although the brewing of *Yamahai‐shikomi* sake with *Yamahai‐shubo* is performed in the subsequent feeding processes (*soe*, *naka*, and *tome*) by adding *koji* and steamed rice with water, it becomes more significant to examine the diversity of bacteria as well as yeasts in the present brewing of *Yamahai‐shikomi* sake with *Yamahai‐shubo* to maintain the quality of the sake (Koyanagi et al., 2016). However, only a limited number of publications have reported on the identification of LAB from *Yamahai‐shubo* samples (Ashizawa & Saito, 1966b; Bokulich et al., 2014; Momose & Kamao., 1993; Sotoike et al., 1964). This is mainly due to there being less information from research works on indigenous microbiota in *Yamahai‐shubo*, and very little is known about the microbial transitions that occur during the preparation of *Yamahai‐shubo*.

With this background, *Pseudomonas* sp. 61‐02 was previously isolated from *Yamahai‐shubo* as a nitrate‐reducing bacterium and determined to be indispensable for the production of *Yamahai‐shikomi* sake (Wakai et al., 1990). Thereafter, we isolated a cocci‐LAB strain LM‐1, a bacilli‐LAB strain LP‐2, and a bacilli‐LAB strain LS‐4 from *Yamahai‐shubo* prepared at Kizakura Brewery, and from the 16 S rRNA sequence and several physiological properties, identified them as *Le. mesenteroides, Lactiplantibacillus plantarum*, and *Ll. sakei*, respectively (Fujiwara et al., 2013). Until now, there has been no publication showing the influence on aromas and tastes in sake produced with *Yamahai‐shubo* using the nitrate‐reducing *Pseudomonas* sp. 61‐02 and three LAB strains. In the present study, we investigated the interactions of four bacterial strains (nitrate‐reducing *Pseudomonas* sp. 61‐02, *Le. mesenteroides* LM‐1, *Lp. plantarum* LP‐2, and *Ll. sakei* LS‐4) in *Yamahai‐shubo* preparation and showed the requirements of two strains, 61‐02 and LM‐1. In the evaluation process, we performed principal component analysis for samples of *Yamahai‐shubo* and *Yamahai‐shikomi sake*. Next, we examined how *Lp. plantarum* LP‐2 and *Ll. sakei* LS‐4 contributed to the quality of sake with the coexistence of *Pseudomonas* sp. strain 61‐02 and *Le. mesenteroides* LM‐1 in *Yamahai‐shikomi* sake brewing. Finally, we showed the significance of *Lp. plantarum* LP‐2 over *Ll. sakei* LS‐4 and demonstrated that the quality of *Yamahai‐shikomi* sake can be controlled by LAB through studying various combinations of those strains in *Yamahai‐shubo*.

## 2 MATERIALS AND METHODS

### 2.1 Microorganisms

Four bacterial strains [nitrate‐reducing *Pseudomonas* sp. 61‐02 and three LAB strains (LM‐1, LP‐2, and LS‐4)] were used in this study. They were previously isolated from *Yamahai‐shubo* described before (Fujiwara et al., 2013; Wakai et al., 1990). The yeast strain, KZ‐06 U, used in this study was a strain of *S. cerevisiae* that was developed for industrial‐scale sake brewing at Kizakura Co., Ltd.

### 2.2 *Yamahai‐shubo* preparation

The initial mixture of *Yamahai‐shubo* was prepared as follows. The mixture included 333 g of total rice [250 g of raw rice (102 g of *koji* and 231 g of steamed rice)] and 255 mL of water with the addition of KNO_3_ (60 mg/L). *koji* is a type of rice that is made from *koji* mold (*A. oryzae* FK‐5, Konnomoyashi Co., Ltd., Hyogo, Japan) with polished rice made by the industrial *koji* manufacturing system (Churitsu Industry Co., Ltd., Tokyo, Japan). Fermentation with *koji* followed the same method described before (Aya, 1995). A polishing ratio of 70% was employed for the raw rice and applied to *koji* and steamed rice.

Four bacterial strains [nitrate‐reducing *Pseudomonas* sp. 61‐02 and three LAB strains (LM‐1, LP‐2, and LS‐4)] were prepared as follows. Strain 61‐02 was independently cultured in PN medium [1% (W/V) polypeptone, 1% (W/V) KNO_3_] for 3 days at 15°C (Wakai et al., 1990) and was adjusted to reach a cell concentration of 10^4^ cells/mL. Three LAB strains were separately cultured in *koji* extract (sake meter −60) for 3 days at 20°C and were added to the initial mixture of *Yamahai‐shubo* at a cell concentration of 10^3^ cells/mL. *Yamahai‐shubo* preparation was totally completed in 25 days with the initial mixture of *Yamahai‐shubo* after adding any of the nitrate‐reducing bacterial strain 61‐02 and/or three LAB bacterial strains (a total of 16 variations depending on the bacterial strain(s) added; see Table 1).

**TABLE 1:** Fermentation factors of “*Yamahai‐shubo”* samples in 25 days after adding any of the nitrate‐reducing bacterial strain 62‐01 and/or three LAB strains (LM‐1, LP‐2, and LS‐4).

No.	Bacterial strain added^a^				SM	Alc % (v/v)	TA^b^ mL‐0.1 N NaOH	AA^b^ mL‐0.1 N NaOH	Glc^c^ % (g/V)	EtAc	iBuAc mg/L	iBuOH mg/L	iAAc mg/L	iAOH mg/L	EtCp mg/L	iAAc/iAOH% (w/w)	Cell number of strain 61‐02^d^ (cells/mL)	Cell number of LABs^d^ (cells/mL)
	61‐02	LM‐1	LP‐2	LS‐4						mg/L								
1	−	−	−	−	−75	12.2	12.6	5.4	9.7	612	0.16	97.1	0.87	126.2	0.06	0.69	n. d.	5.0 × 10^3^
2	−	+	−	−	−139	5.3	14.6	5.7	19.2	925	0.05	1.4	0.33	35.0	0.01	0.95	n. d.	2.6 × 10^6^
3	−	−	+	−	−62	12.0	14.8	4.4	7.4	206	0.12	118.6	0.72	134.6	0.09	0.54	n. d.	n. d.
4	−	−	−	+	−75	12.2	11.6	5.6	9.5	695	0.21	106.6	1.18	129.3	0.08	0.92	n. d.	n. d.
5	−	+	+	−	−60	14.0	13.4	4.5	7.1	248	0.14	125.2	0.74	142.6	0.12	0.52	n. d.	3.0 × 10^3^
6	−	+	−	+	−75	12.5	10.7	5.5	9.5	576	0.17	102.0	0.95	128.0	0.08	0.74	n. d.	n. d.
7	−	−	+	+	−76	12.2	11.6	5.7	8.8	720	0.23	105.5	1.27	126.3	0.12	1.01	n. d.	n. d.
8	−	+	+	+	−75	12.0	11.7	5.6	9.1	690	0.21	96.7	1.19	124.9	0.12	0.96	n. d.	6.0 × 10^3^
9	+	−	−	−	−71	12.9	9.3	6.4	8.8	495	0.22	105.4	1.04	117.9	0.14	0.88	n. d.	n. d.
10	+	+	−	−	−148	4.1	9.5	7.5	19.1	1198	0.22	16.7	0.98	43.1	0.24	2.28	n. d.	4.4 × 10^7^
11	+	−	+	−	−54	14.3	13.4	4.3	6.3	109	0.12	116.5	0.77	136.8	0.23	0.56	n. d.	5.6 × 10^2^
12	+	−	−	+	−64	13.6	9.7	4.9	7.4	56	0.16	90.0	0.99	119.0	0.30	0.83	n. d.	n. d.
13	+	+	+	−	−57	14.4	12.1	4.8	6.6	36	0.05	62.1	0.33	80.6	0.18	0.41	n. d.	n. d.
14	+	+	−	+	−75	12.4	10.1	4.5	9.1	335	0.20	82.1	1.03	104.3	0.22	0.98	n. d.	n. d.
15	+	−	+	+	−62	13.8	10.3	5.0	7.4	47	0.06	29.6	0.40	57.0	0.21	0.70	n. d.	n. d.
16	+	+	+	+	−67	13.5	10.6	6.0	7.5	108	0.16	84.7	0.89	111.7	0.36	0.79	n. d.	n. d.

*Note*: iAAc/iAOH. These fermentation factors were determined by the standard methods established by the National Tax Agency of Japan (National Research Institute of Brewing, 2017).

Abbreviations: AA, amino acidity; Alc, ethanol; EtAc, ethyl acetate; EtCp, ethyl caproate; iAAc, isoamyl acetate; iAOH, isoamyl alcohol; iBuAc, isobutyl acetate; iBuOH, isobutyl alcohol; n.d., not detected; SM, sake meter; TA, total acidity.

^a^ The bacteria added for *Yamahai‐shubo* preparation were indicated with + and those that were not added were indicated with −.

^b^ TA and AA are expressed as the amount of volume required to neutralize with 0.1 N NaOH solution in 10 mL of *Yamahai‐shubo* samples.

^c^ Glucose (Glc) was measured by the method described previously (Fujiwara et al., 2013) using a specific instrument (Arkray ADAMS Glucose GA‐1151, Kyoto, Japan).

^d^ The cell numbers of strain 61‐02 and LABs (strains LM‐1, LP‐2, and LS‐4) were determined by the method described previously (Fujiwara et al., 2013; Wakai et al., 1990).

The temperature during *Yamahai‐shubo* preparation was kept at 8°C until day 4. After day 5, the preparation was warmed up by contact with hot water at 65°C to increase the temperature by one degree per day until it reached 21°C on day 16. After holding the temperature of the mixture at 21°C for another 5 days, the temperature was decreased to 15°C on day 21, to 13°C on day 22, to 12°C on day 23, and was then maintained at 12°C until day 25. The yeast strain KZ‐06 U was separately cultured in *koji* extract (sake meter −60) for 2 days at 30°C and added between days 10 and 15 of the preparation to reach a cell concentration of 10^6^ cells/mL when the nitrite concentration of the *Yamahai‐shubo* preparation was less than 1.0 mg/L. The timing of yeast addition in the absence of strain 61‐02 was the same as the timing of yeast addition in the presence of strain 61‐02. The final preparation reached approximately 500 mL in volume and was called “*Yamahai‐shubo*.” For use at the industrial scale, the process was considered complete at day 25, even if the disappearance of nitrite was delayed and the yeast growth period was shortened.

The cell numbers of strain 61‐02 and three LAB strains (LM‐1, LP‐2, and LS‐4) were determined by the method described previously (Fujiwara et al., 2013; Wakai et al., 1990).

### 2.3 *Yamahai‐shikomi* sake brewing

The polishing ratio of raw rice was set at 70%, just as in the case for *Yamahai‐shubo* preparation. Brewage ingredients (*Yamahai‐shubo*, *koji*, steamed rice, and water) were mixed at three different times (*soe*, *naka*, and *tome* feeding procedures) to smoothly complete the fermentation (Anzawa et al., 2014; Furukawa et al., 2000). The *soe* procedure (23 mL of *Yamahai‐shubo*, 9.6 g of *koji*, 26.6 g of steamed rice, and 33 mL of water were added) was carried out at 15°C from day 1 to day 2. Both the *naka* (day 3, 12.0 g of *koji*, 54.6 g of steamed rice, and 64 mL of water) and the *tome* (day 4, 14.4 g of *koji*, 99.4 g of steamed rice, and 112 mL of water) feeding procedures were carried out by adding the components at 10°C. After the *tome* procedure (170 g of raw rice, total volume 450 mL), sake brewing was monitored by measuring the weight reduction of the fermentation mixture, corresponding to CO_2_ evolution for 25 days. The temperature of the fermentation mixture was increased by about 0.5°C for another 6 days, kept at 13°C for an additional 7 days, and then promptly lowered to 10°C while the temperature was maintained for an additional 12 days. After sake brewing (25 days), the samples were separated by centrifugation (6800 × *g*, 20 min, 4°C), and the supernatants were called “*Yamahai‐shikomi*” sake samples (a total of 16 types of *Yamahai‐shikomi* sake samples depending on 16 variations of *Yamahai‐shubo*; see Table 2). The volume of sake and the weight of sake lee were measured. Sake lees are the final sediments obtained in the sake brewing process and are alternatively called “sake‐kasu.”

**TABLE 2:** Fermentation factors in “*Yamahai‐shikomi*” sake samples brewed with various “*Yamahai‐shubo*” after 25 days.

No.	Bacterial strain added to “*Yamahai‐shubo*”^a^				Sake Vol. (mL)	Sake Lee (g)	Sake Lee/sake Vol.	SM	Alc % (v/v)	TA^b^ mL‐0.1 N NaOH	AA^b^ mL‐0.1 N NaOH	Glc^c^ % (w/v)	UV		EtAc mg/L	iBuAc mg/L	iBuOH mg/L	iAAc mg/L	iAOH mg/L	EtCp mg/L	iAAc/iAOH% (w/w)	Sensory score^d^	
	61‐02	LM‐1	LP‐2	LS‐4									260 Nm	280 Nm								Aroma	Taste
1	−	−	−	−	272	106	0.39	−11.9	17.9	3.1	2.3	1.4	11.4	10.0	45	0.07	41.3	3.0	142	0.34	2.09	2.7	2.5
2	−	+	−	−	256	125	0.49	−17.3	16.6	5.4	2.8	1.7	11.1	10.5	41	0.01	15.6	1.0	113	1.64	0.91	3.3	3.5
3	−	−	+	−	266	118	0.45	−12.9	17.3	3.3	2.1	1.4	10.5	9.7	58	0.10	47.6	3.9	158	0.53	2.49	2.3	2.3
4	−	−	−	+	273	113	0.41	−9.9	18.3	3.0	2.1	1.3	10.8	9.8	78	0.15	56.8	5.2	173	0.61	3.00	2.8	2.8
5	−	+	+	−	274	110	0.40	−11.0	18.0	3.2	2.1	1.4	10.9	9.9	47	0.07	32.5	3.1	125	0.43	2.51	2.3	2.7
6	−	+	−	+	286	99	0.35	−8.7	18.2	3.1	2.3	1.3	11.7	10.2	53	0.09	54.5	3.7	168	0.50	2.18	3.0	2.7
7	−	−	+	+	279	106	0.38	−10.6	18.0	3.0	2.2	1.3	10.9	9.8	75	0.14	56.0	5.0	172	0.61	2.94	3.0	2.8
8	−	+	+	+	270	115	0.43	−12.9	17.3	3.0	2.0	1.3	10.4	9.7	53	0.09	43.3	3.5	152	0.53	2.31	2.8	2.5
9	+	−	−	−	261	120	0.46	−14.1	17.2	3.0	1.9	1.4	10.5	9.8	55	0.12	46.0	4.1	153	0.63	2.69	3.0	2.7
10	+	+	−	−	266	115	0.43	−16.5	16.7	3.9	2.4	1.5	10.7	9.9	53	0.05	37.2	2.1	137	0.42	1.51	2.7	2.8
11	+	−	+	−	274	112	0.41	−11.7	17.7	3.1	2.0	1.4	10.6	9.7	68	0.13	47.8	4.7	160	0.70	2.92	2.3	2.3
12	+	−	−	+	285	99	0.35	−9.9	18.2	3.0	2.0	1.4	11.9	10.3	80	0.18	58.1	5.9	174	0.71	3.36	2.5	2.3
13	+	+	+	−	279	105	0.37	−11.6	17.8	3.2	2.0	1.4	11.4	10.3	65	0.13	50.1	4.4	162	0.66	2.69	2.3	2.5
14	+	+	−	+	268	116	0.43	−12.9	17.8	3.0	1.9	1.4	10.8	9.8	80	0.17	52.0	5.4	167	0.74	3.23	2.5	2.7
15	+	−	+	+	267	116	0.43	−13.4	17.5	3.1	1.9	1.3	10.4	9.8	54	0.12	45.3	4.1	158	0.71	2.58	2.5	2.7
16	+	+	+	+	280	103	0.37	−10.8	18.1	3.1	2.0	1.4	10.9	9.8	86	0.18	49.4	5.9	168	0.84	3.49	2.2	2.5

*Note*: iAAc/iAOH. These fermentation factors were determined by the standard methods established by the National Tax Agency of Japan (National Research Institute of Brewing, 2017).

Abbreviations: AA, amino acidity; Alc, ethanol; EtAc, ethyl acetate; EtCp, ethyl caproate; iAOH, isoamyl alcohol; iBuAc, isobutyl acetate; iBuOH, isobutyl alcohol; SM, sake meter; TA, total acidity; UV, Ultraviolet absorption.

^a^ The bacteria added for *Yamahai‐shubo* preparation were indicated with + and those that were not added were indicated with ‐. The patterns of strains added to *Yamahai‐shubo* are the same as those in Table 1.

^b^ TA and AA are expressed as the amount of volume required to neutralize with 0.1 N NaOH solution in 10 mL of *Yamahai‐shikomi* sake samples.

^c^ Glucose (Glc) was measured by the method described previously (Fujiwara et al., 2013) using a specific instrument (Arkray ADAMS Glucose GA‐1151, Kyoto, Japan).

^d^ The sensory scores of sake samples held at 20°C consisted of qualitative evaluations of aromas and tastes according to five different grades (1, very good; 2, good; 3, normal; 4, bad; 5, very bad). Sensory evaluations were performed by six well‐trained panelists.

### 2.4 Analysis of fermentation factors in *Yamahai‐shubo* preparation and sake samples

Basic fermentation factors for sake meter (SM), ethanol (Alc), total acidity (TA), amino acidity (AA), and Ultraviolet absorption (UV) were determined by the standard method established by National Research Institute of Brewing (National Research Institute of Brewing, 2017), while glucose (Glc) was measured by the method described previously (Fujiwara et al., 2013) using a specific instrument (Arkray ADAMS Glucose GA‐1151, Kyoto, Japan). Fermentation factors for volatile organic compounds [ethyl acetate (EtAc), isobutyl acetate (iBuAc), isobutyl alcohol (iBuOH), isoamyl acetate (iAAc), isoamyl alcohol (iAOH), ethyl caproate (EtCp), and isoamyl acetate/isoamyl alcohol (iAAc/iAOH)] were determined by following the same standard methods (National Research Institute of Brewing, 2017). Other fermentation factors, including concentrations of other organic acids (pyruvic acid, citric acid, succinic acid, malic acid, and lactic acid), were determined by the method described previously (Kohsaka et al., 2016): 25 μL of sample was taken in a 10 mL screw‐top test tube, and 1 mL of 0.3 M HCl/1‐propanol solution was added. It was heated at 100°C for 1 h. After cooling, 1 mL of methyl *tert*‐butyl ether containing 4 μg of methyl laurate as an internal standard and 1 mL of distilled water were added and mixed. The ether layer was analyzed by a gas chromatography GC‐2014 (Shimazu Corporation, Kyoto, Japan). Diacetyl was derivatized by Shinwa DS‐DA (Shinwa Chemical Industries, Ltd., Kyoto, Japan, https://shinwa‐cpc.co.jp/products/ds/) and analyzed using the same gas chromatography system. All data are the averages of two or three independent experiments.

The qualities of *Yamahai‐shikomi* sake samples were graded by sensory scores evaluated by four or six well‐trained panelists. The sensory scores of sake samples held at 20°C consisted of qualitative evaluations of aromas and tastes according to five different grades (1, very good; 2, good; 3, normal; 4, bad; 5, very bad).

### 2.5 Principal component analysis (PCA)

Principal component analysis (PCA) was performed with Excel Statistical analysis 2008 software for Windows (Social Survey Research Information Co., Ltd., https://www.bellcurve.jp/), employing fermentation factors (SM, Alc, TA, AA, Glc, UV, EtAc, iBuAc, iBuOH, iAAc, iAOH, EtCp, and iAAc/iAOH) and sensory scores at the final point (25 days) of *Yamahai‐shubo* (Table 1) and *Yamahai‐shikomi* sake samples (Table 2). In addition, the data for sake volume and sake lee, sake lee/sake volume, were used for the analysis.

## 3 RESULTS

### 3.1 Effect of four bacterial strains (nitrate‐reducing *pseudomonas* sp. 61‐02, *Le. Mesenteroides* LM‐1, *Lp. Plantarum* LP‐2, and *Ll. Sakei* LS‐4) on *Yamahai‐shubo* preparation samples

**FIGURE 1:**
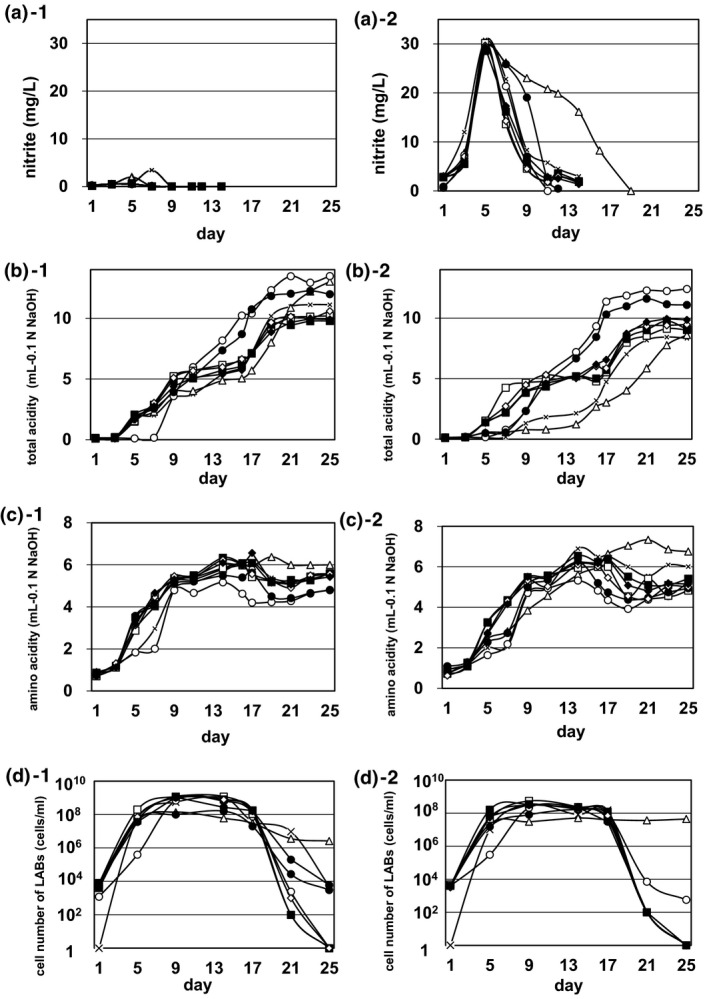
Changes of fermentation factors in *Yamahai‐shubo* preparation samples along the time course for 25 days; nitrite (a‐1, a‐2), total acidity (b‐1, b‐2), amino acidity (c‐1, c‐2), cell number of LABs (strains LM‐1, LP‐2, and LS‐4; d‐1, d‐2). The *Yamahai‐shubo* preparation was carried out as in Materials and Methods and completed in totally 25 days with the initial mixture for *Yamahai‐shubo* after adding any of three LAB bacterial strains (LM‐1, LP‐2, and LS‐4) with (a‐2, b‐2, c‐2, d‐2) and without (a‐1, b‐1, c‐1, d‐1) the nitrate‐reducing bacterium strain No. 61‐02. Those determinations were done in duplicate, and the average values were indicated in the figures. Cross (×), no LABs; open triangle (Δ), LM‐1 strain; open circle (○), LP‐2; open square (□), LS‐4; closed circle (●), LM‐1 and LP‐2; closed square (■), LM‐1 and LS‐4; open diamond (◊), LP‐2 and LS‐4; closed diamond (♦), LM‐1, LP‐2, and LS‐4.

**FIGURE 2:**
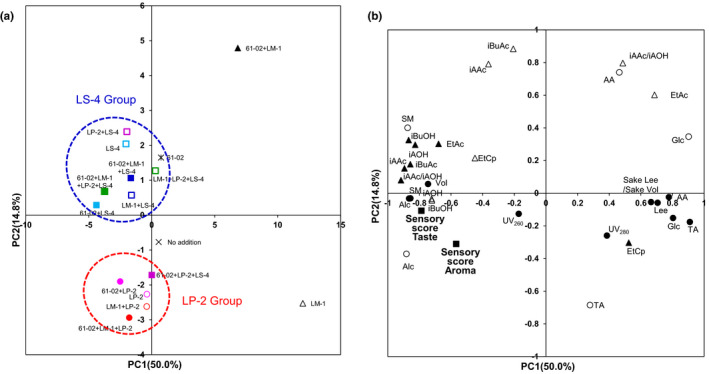
Principal component analysis (PCA) for *Yamahai‐shubo* preparation and *Yamahai‐shikomi* sake. (a) Score plot. Contribution ratios of PC1 and PC2 were 50.0% and 14.8%, respectively. Combination of addition of the strain No.61‐02 and three LAB strains (LM‐1, LP‐2, and LS‐4) to *Yamahai‐shubo* preparation and *Yamahai‐shikomi* sake: No addition (×), no addition of any strains (No. 1 in Tables 1 and 2); LM‐1 (Δ), addition of the strain LM‐1 (No. 2); LP‐2 (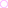), addition of the strain LP‐2 (No. 3); LS‐4 (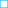), addition of the strain LS‐4 (No. 4); LM‐1 + LP‐2 (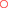), addition of the strains LM‐1 and LP‐2 (No. 5); LM‐1 + LS‐4 (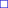), addition of the strains LM‐1 and LS‐4 (No. 6); LP‐2 + LS‐4 (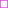), addition of the strains LP‐2 and LS‐4 (No. 7); LM‐1 + LP‐2 + LS‐4 (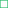), addition of the strains LM‐1, LP‐2, and LS‐4 (No. 8); 61‐02 (*), addition of 61‐02 (No. 9); 61‐02 + LM‐1 (▲), addition of strain No.61‐02 and LM‐1 (No. 10); 61‐02 + LP‐2 (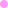), addition of the strains 61‐02 and LP‐2 (No. 11); 61‐02 + LS‐4 (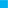), addition of the strains 61‐02 and LS‐4 (No. 12); 61‐02 + LM‐1 + LP‐2 (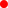), addition of the strains 61‐02, LM‐1, and LP‐2 (No. 13); 61‐02 + LM‐1 + LS‐4 (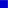), addition of the strains 61‐02, LM‐1, and LS‐4 (No. 14); 61‐02 + LP‐2 + LS‐4 (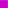), addition of the strains 61‐02, LP‐2, and LS‐4 (No. 15); 61‐02 + LM‐1 + LP‐2 + LS‐4 (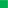), addition of the strains 61‐02, LM‐1, LP‐2, and LS‐4 (No. 16). (b) Loading plot of the samples of *Yamahai‐shubo* preparation and *Yamahai‐shikomi* sake are shown in Tables 1 and 2. Thirty‐one fermentation factors were obtained as shown in Materials and Methods. Open symbols: those data were from *Yamahai‐shubo* samples. Closed symbols: those data were from *Yamahai‐shikomi* sake samples. Circle; basic fermentation factors (SM, sake meter; Alc, ethylalcohol; TA, total acidity; AA, amino acidity; Glc, glucose concentration; UV260, Ultraviolet absorption at 260 nm; UV280, Ultraviolet absorption at 280 nm; Vol, sake volume; Lees; sake lees; Lee/Vol, sake lees/sake volume). Triangle; fermentation factors in aroma (EtAc, ethyl acetate; iBuAc, isobutyl acetate; iBuOH, isobutyl alcohol; iAAc, isoamyl acetate; iAOH, isoamyl alcohol; EtCp, ethyl caproate; iAAc/iAOH). Square; sensory scores in aroma and taste for *Yamahai‐shikomi* sake.

The four bacterial strains previously found in *Yamahai‐shubo* were examined to evaluate their effects along the time course of *Yamahai‐shubo* preparation (a total of 25 days). Changes in three fermentation factors are depicted in Figure 1, and others are shown in Table 1. In Figure 1, three factors—nitrite concentration (a‐1 and a‐2), acidity (b‐1 and b‐2), and amino acid concentration (c‐1 and c‐2)—were described in the absence (a‐1, b‐1, and c‐1) and presence (a‐2, b‐2, and c‐2) of nitrate‐reducing *Pseudomonas* sp. 61‐02, following the method as described in Materials and Methods. Nitrite is known to be essential in *Yamahai‐shubo* preparation process; it is converted from nitrate, which is derived from PN medium for the preparation of strain 61‐02 (see Materials and Methods) and inhibits the growth of wild yeasts that are concomitant in the initial mixture for *Yamahai‐shubo*. As shown in Figure 1a‐1,a‐2, strain 61‐02 was responsible for nitrite production; its concentration increased in the first half of *Yamahai‐shubo* preparation and drastically decreased before the second half. However, the coculture of strain LM‐1 only with strain 61‐02 was found to retard the decrease in the concentration of nitrite (Figure 1a‐2). It is noted that the delay is a characteristic of strain LM‐1 when it is cocultured with strain 61‐02. The acidity increased along the time course of *Yamahai‐shubo* preparation in the absence and presence of strain 61‐02, which is necessary for the growth of yeast in sake brewing of strain KZ‐06 U; on the other hand, there was a slower rise in acidity for the coculture of strain LM‐1 only with strain 61‐02 and the simple culture of strain 61‐02 without any LAB strains (Figure 1b‐1,b‐2). Furthermore, there seemed to be no remarkable difference in amino acid concentration in the presence and absence of strain 61‐02 (Figure 1c‐1,c‐2). In addition, with the exceptions of *Yamahai‐shubo* preparations contained strains 61‐02 and LM‐1 (No. 10 in Table 1) and only strain LM‐1 (No. 2 in Table 1), the viable cell numbers of four bacterial strains (61‐02, LM‐1, LP‐2, and LS‐4) in *Yamahai‐shubo* became extremely decreased after the preparation for 25 days (Figure 1d‐1,d‐2 and Table 1). In the first half of the preparation of *Yamahai‐shubo*, there seemed to be no significant difference in cell number of LABs (strains LM‐1, LP‐2, and LS‐4) in the presence and absence of strain 61‐02 (Figure 1d‐1,d‐2). However, *Yamahai‐shubo* preparations contained only strain 61‐02 (No. 9 in Table 1) and no addition of any strains (No. 1 in Table 1) had no LABs on day 1, but they increased LABs on day 5. Although the growth of LABs is confirmed in the industrial scale *Yamahai‐shubo* preparation without the addition of microorganisms (Ashizawa & Saito, 1966a; Koyanagi et al., 2016), it is known that the process is not stable at the industrial scale. On the other hand, we have previously confirmed that *Yamahai‐shubo* can be stably incubated at an industrial scale by adding cocci LAB and bacilli LAB (Fujiwara et al., 2013) without being affected by environmental microorganisms, and therefore, as a negative control, we used *Yamahai‐shubo* preparations contained only strain 61‐02 and no addition of any strains. After yeast strain KZ‐06 U was added in *Yamahai‐shubo* preparation between day 11 and day 19, alcohol concentration changes were almost the same at the 16 patterns (data not shown).

Comparing the components at the final point (25 days) of *Yamahai‐shubo* preparation in Table 1, the addition of strain LP‐2 (Nos. 5 and 11) contributed to the highest level of the alcohol concentration (Alc) and total acidity (TA), simultaneously resulting in essential requirements of strains 61‐02 and/or LM‐1 for *Yamahai‐shubo* utilizing the advantages of strain LP‐2 without strain LS‐4.

We further investigated fermentation factors for *Yamahai‐shikomi* sake similarly as *Yamahai‐shubo* (Table 2) and applied them to principal component analysis (PCA).

### 3.2 Principal component analysis (PCA) for *Yamahai‐shubo* and *Yamahai‐shikomi* sake

To investigate the suitability of the bacterial combination of the nitrate‐reducing *Pseudomonas* sp. 61‐02 and three LAB strains (LM‐1, LP‐2, and LS‐4), we performed principal component analysis (PCA) by using fermentation factors for 16 samples of *Yamahai‐shubo* and *Yamahai‐shikomi* sake, as shown in Tables 1 and 2. As a result of PCA score plotting of fermentation factors, the contribution ratios of principal component 1 (PC1) and principal component 2 (PC2) were 50.0% and 14.8%, respectively (Figure 2). In addition, the eigen values of principal component 1 (PC1) and principal component 2 (PC2) were 15.5 and 4.6, respectively.

In the score plot (Figure 2a), when *Yamahai‐shubo* preparations and *Yamahai‐shikomi* sake samples contained strains 61‐02 and LM‐1 (No. 10 in Tables 1 and 2) or only strain LM‐1 (No. 2), their plots were located in the first and fourth quadrants, respectively, apart from other combinations of strains that occurred in the second and third quadrants. In the second and third quadrants, two major groups were formed. Plots for the group containing strain LP‐2 (LP‐2 group; Nos. 3, 5, 11, 13, and 15) occurred in the third quadrants, forming one cluster. In contrast, plots for the group including strain LS‐4 (LS‐4 group; Nos. 4, 6, 7, 12, and 14) occurred in the second quadrant. The sample only contained strain 61‐02 (No. 9) was located in LS‐4 group. Interestingly, combinations of three LAB strains (LM‐1, LP‐2, and LS‐4) with (No. 16) and without strain 61‐02 (No. 8) were included in this LS‐4 group. These results showed that the characteristics of strain LS‐4 in the LS‐4 group were predominant in the second quadrant when strain LP‐2 was present with strain LM‐1.

In order to evaluate the fermentation factors that contribute to the principal component scores, we confirmed the factor loadings by their plots (Figure 2b). The factor loadings are the correlation coefficients between the principal components and the variable fermentation factors. The closer the values of factor loading are to 1 or −1, the more the factors contribute to the principal components. On the PC1 axis, the factor loading of TA for *Yamahai‐shikomi* sake was 0.91. On the other hand, the factor loading of the sensory score for taste showed a negative value of −0.79. On the PC2 axis, the factor loadings of iBuAc for *Yamahai‐shubo* and of EtAc for *Yamahai‐shikomi* sake were 0.88 and 0.30, respectively. Since EtAc and iBuAc generally provide aromas undesirable for sake products (Yoshizawa, 1999), their positive values are disadvantageous in the quality of sake. In contrast, the factor loading of the sensory score for aroma of *Yamahai‐shikomi* sake showed a negative value of −0.31 (Figure 2b). The factor loading plots showed that negative values of sensory evaluation contributed to the principal components of the PC1 and PC2 axes (Figure 2b). In the score plot, since the LP‐2 group showed negative values on the PC1 and PC2 axes (third quadrant, Figure 2a), strain LP‐2 was confirmed to be responsible for a group with a higher sensory score. On the other hand, the sample containing strain 61‐02 and LM‐1 (No. 10) showed positive values on the PC1 and PC2 axes (first quadrant, Figure 2a). EtAc and iAAC/iAOH for *Yamahai‐shubo* were both located in the first quadrant (Figure 2b). Although iAAC/iAOH is the indicator of the good quality of sake, EtAc for *Yamahai‐shubo* may decrease sake quality while iAAC/iAOH for *Yamahai‐shubo* may be less effective in improving sake quality.

Judging from these results, it was found that strains LP‐2 and LS‐4 were important in the *Yamahai‐shikomi* sake in the presence of strains 61‐02 and LM‐1.

### 3.3 Effect of *Lp. Plantarum* LP‐2 and *Ll. Sakei* LS‐4 in *Yamahai‐shubo* preparation on the concentration of organic acids in *Yamahai‐shikomi* sake

**FIGURE 3:**
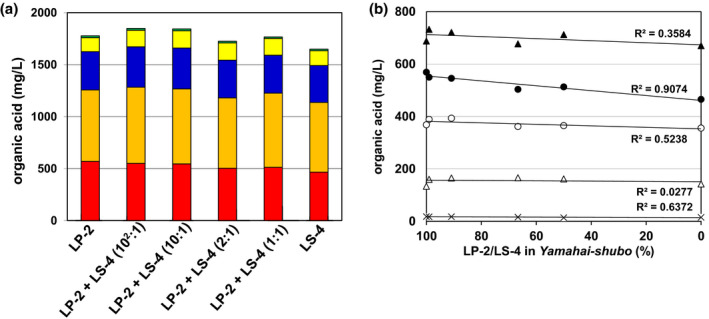
Concentrations (mg/L) of organic acids (pyruvic acid, citric acid, succinic acid, malic acid, and lactic acid) produced in *Yamahai‐shikomi* sake samples (a) and change of each acid relative to the proportion of LP‐2 to LS‐4 strains occurring in *Yamahai‐shubo* (b). (a) The concentrations were indicated with (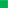) for pyruvic acid, (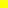) for citric acid, (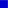) for succinic acid, (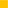) for malic acid, and (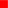) for lactic acid. *Yamahai‐shikomi* sake was obtained by the method as described in Materials and Methods with different *Yamahai‐shubo* samples prepared by adding various proportions of strains LP‐2 and LS‐4 to its initial mixture of *Yamahai‐shubo* with the strains 61‐02 and LM‐1. (b) The change of each acid [(×) pyruvic acid, (Δ) citric acid, (▲) succinic acid, (○) malic acid, and (●) lactic acid] relative to the proportion of LP‐2 to LS‐4 strains occurring in *Yamahai‐shubo*. The dots on the line for each organic acid correspond to LP‐2, LP‐2 + LS‐4 (10^2^:1), LP‐2 + LS‐4 (10:1), LP‐2 + LS‐4 (2:1), LP‐2 + LS‐4 (1:1), and LS‐4 in Figure 2 a from left to right. The values of R^2^ represent the correlation coefficients.

The sample coexisting strains LP‐2 and LS‐4 was located in the LS‐4 group (Figure 2a). So, to evaluate the effect of coexisting bacilli‐LABs on sake quality, *Yamahai‐shubo* samples were prepared by adding different proportions of strains LP‐2 and LS‐4 to its initial mixture in the presence of conventional amounts of strains 61‐02 and LM‐1. After sake brewing with those *Yamahai‐shubo* samples for 25 days, the *Yamahai‐shikomi* sake samples were withdrawn and prepared by following the method as described in the Materials and Methods section, and then concentrations of organic acids (pyruvic acid, citric acid, succinic acid, malic acid, and lactic acid) in *Yamahai‐shikomi* sake and *Yamahai‐shubo* samples were determined for each sample (Figure 3). Although the sake samples were produced in the same way as those *Yamahai‐shubo* samples including bacterial strains with different proportions of strains LP‐2 and LS‐4, there seemed to be no significant difference in the concentration of five organic acids (Figure 3a). However, taking into account the change in each acid relative to the proportion of strains LP‐2 to LS‐4 strains in *Yamahai‐shubo* preparation, only lactic acid was found to decrease with an adequate correlation to the decrease in the ratio of LS‐4 strain (R^2^ = 0.9074; Figure 3b). Although the proportion of strains LP‐2 and LS‐4 was unequal, there seems to be a tendency for lactic acid concentration to increase as the mixing ratio of strain LS‐4 decreases.

### 3.4 Effect of *Lp. Plantarum* LP‐2 and *Ll. Sakei* LS‐4 in *Yamahai‐shubo* preparation on the concentration of diacetyl in *Yamahai‐shikomi* sake

**FIGURE 4:**
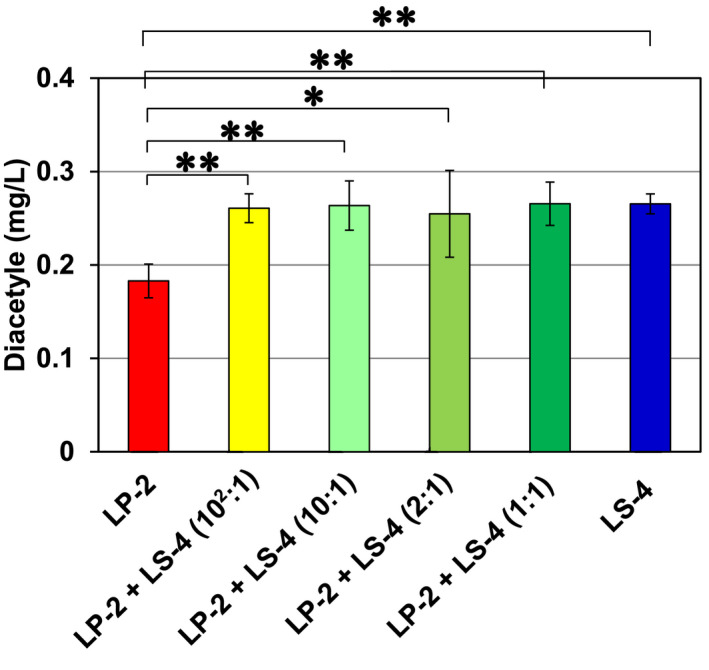
Effects of strains LP‐2 and LS‐4 on the concentrations (mg/L) of diacetyl in *Yamahai‐shikomi* sake. *Yamahai‐shubo* was prepared by adding various proportions of strains LP‐2 and LS‐4 to its initial mixture of *Yamahai‐shubo* with the strains 61‐02 and LM‐1. Six different proportions of strains LP‐2 and LS‐4 added were as follows; LP‐2 only (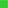), LP‐2:LS‐4 = 10^2^: 1 (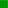), LP‐2:LS‐4 = 10: 1 (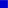), LP‐2:LS‐4 = 2: 1 (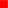), LP‐2:LS‐4 = 1: 1 (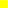), LS‐4 only (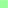). After the sake brewing with seven types of *Yamahai‐shubo*, *Yamahai‐shikomi* sake samples were withdrawn and prepared by following the method as described in Materials and Methods and then concentrations of diacetyl were determined by the method described in Materials and Methods. The data were represented with error bars from the average of the three data. The Student's *t*‐test was used for statistical analysis. **p < .01; *p < .05.

**FIGURE 5:**
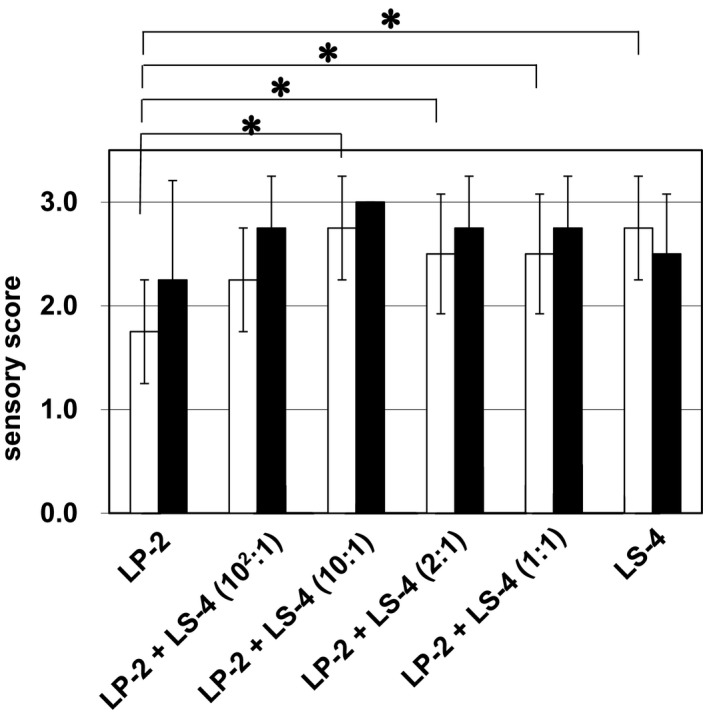
Sensory scores for aroma (□) and taste (■) of *Yamahai‐shikomi* sake samples produced with different proportions of strains LP‐2 and LS‐4 added to the initial mixture of *Yamahai‐shubo* with the strains 61‐02 and LM‐1. The sensory scores of sake samples held at 20°C consisted of qualitative evaluations of aromas and tastes according to five different grades (1, very good; 2, good; 3, normal; 4, bad; 5, very bad). Sensory evaluations were performed by four well‐trained panelists. The Student's *t*‐test was used for statistical analysis. *p < .05. Sensory score values represent mean ± SD (*n* = 4).

We investigated their effect on the concentration of diacetyl. The sake brewing was carried out in the same way as shown in Figure 3, and the concentrations of diacetyl in *Yamahai‐shikomi* sake samples were determined. As shown in Figure 4, the sample prepared in the absence of strain LS‐4 significantly exhibited the lowest concentration of diacetyl. Lower concentrations of diacetyl are so favorable for improving sake quality that the use of strain LS‐4 in *Yamahai‐shubo* preparation had an undesirable effect on the final sake samples. Although diacetyl concentration of LP2 + LS4 (10^2^:1) was higher than that of LP2 + LS4 (2:1), there was no significant difference between them (Figure 4). This result was supported by the statistical analysis for the sensory scores for aroma of each *Yamahai‐shikomi* sake sample (Figure 5). The sensory scores for aroma showed that *Yamahai‐shikomi* sake prepared with strain LP‐2 had significantly lower values than those with strain LS‐4, with the exception of the case with a proportion of LP‐2:LS‐4 = 10^2^:1.

Through this investigation, in order to reduce the diacetyl concentration in *Yamahai‐shikomi* sake, it was found that the addition of strain LS‐4 should be avoided in *Yamahai‐shubo* preparation, and it is likely that strain LP‐2 plays a more significant role in reducing the concentration of diacetyl with strains LM‐1 and 61‐02 in *Yamahai‐shubo* preparation and *Yamahai‐shikomi* sake brewing.

## 4 DISCUSSION

The steady supply of *Yamahai‐shikomi* sake employing *Yamahai‐shubo* has been requested by many sake producers and consumers, while it requires various developments in technological and scientific fields. Therefore, the use of microorganisms (yeasts and bacteria) isolated from *Yamahai‐shubo* samples is very important for producing *Yamahai‐shikomi* sake. In particular, the isolation of different bacteria from *Yamahai‐shubo* samples and their practical use in sake brewing make it possible to produce *Yamahai‐shikomi* sake under artificial control in the recent process.

*Yamahai‐shubo* samples contain various kinds of identified and unidentified bacteria, and their bacterial diversities vary depending on the sources from which they are derived. It is well known that the quality of *Yamahai‐shikomi* sake depends on the diversities of those bacteria. Therefore, it is necessary to grow advantageous bacteria to prepare appropriate *Yamahai‐shubo* samples and to achieve the desired sake quality.

We previously isolated four bacterial strains (nitrate‐reducing *Pseudomonas* sp. 61‐02, cocci‐LAB *Le. mesenteroides* LM‐1, bacilli‐LAB *Lp. plantarum* LP‐2, and bacilli‐LAB *Ll. sakei* LS‐4) from the *Yamahai‐shubo* sample (Fujiwara et al., 2013; Wakai et al., 1990). In our previous report, we confirmed that strain LP‐2 is industrially available, but it remained unconfirmed how the *Yamahai‐shubo* preparation and the *Yamahai‐shikomi* sake brewing change under the coexistence of strains LP‐2 and LS‐4 (Fujiwara et al., 2013). In the present study, we confirmed that the nitrate‐reducing strain 61‐02 was necessary for sufficient nitrite production, whereas the decrease in nitrite was delayed where only the cocci‐LAB strain LM‐1 was present with the nitrate‐reducing strain 61‐02 (Figure 1a‐2). Sato et al. cultivated lactic acid bacteria by adding nitrite to two different types of culture media and confirmed that the reduction in nitrite was closely linked to the decrease in the pH of the culture (Sato et al., 2016). They attributed the decrease in the pH of the media to lactic acid produced by LAB, resulting in a fall in nitrite due to a nonenzymatical diazotization reaction with various amino groups occurring in the environment (Yoshizawa, 1999). They also reported that the reduction in nitrite was more retarded by the presence of *Le. mesenteroides* than that of *Ll. sakei*, since the production of lactic acid was much slower in the presence of *Le. mesenteroides* than of *Ll. sakei*. It indicates that the reduction in nitrite is delayed only in a cocci‐LAB strain (*Le. mesenteroides*) that produces lactic acid more slowly. We clarified the turnover of those bacteria, along with the amount of nitrite, through this study.

In *Yamahai‐shubo* samples after 25 days (Table 1), the addition of strain LP‐2 provided higher acidity than the addition of LS‐4 in the presence of strains 61‐02 and LM‐1 (see the results of Nos. 13 and 14). It has already been confirmed that the acid production of strain LP‐2 in *Yamahai‐shubo* is higher than that of strain LS‐4 (Fujiwara et al., 2013). At the same reason, the addition of strain LP‐2 provided higher acidity than the addition of LS‐4 (see the results of Nos. 11 and 12) in the presence of strain 61‐02 and the absence of stain LM‐1. After sake brewing with the *Yamahai‐shubo* samples, the acidity of *Yamahai‐shikomi* sake samples when strain LP‐2 was added to *Yamahai‐shubo* was higher than that when strain LS‐4 was added (see TA of Nos. 13 and 14 in Table 2). Since the volume of *Yamahai‐shubo* accounted for only 5% of the total volume of *Yamahai‐shikomi* sake after the *tome* procedure (23 mL‐*Yamahai‐shubo*/450 mL‐total volume; see Materials and Methods), *Yamahai‐shubo* was diluted nearly 20 times in *Yamahai‐shikomi* sake. Thus, the higher acidity of *Yamahai‐shubo* was supposed to be unable to directly keep the acidity of *Yamahai‐shikomi* sake. On the other hand, the majority of the acids in *Yamahai‐shikomi* sake were most likely produced by yeasts coexisting during *Yamahai‐shikomi* sake brewing under higher acidic environment, resulting in increased acidity. This was consistent with the fact that the viable cell numbers of most bacteria in *Yamahai‐shubo* became extremely decreased after 25 days (Table 1); therefore, acids produced by the bacteria de novo during the brewing of *Yamahai‐shikomi* sake was negligible.

In Figure 2, the LP‐2 group showed preferable sensory score values for aroma and taste. Diacetyl (2,3‐butanedione) is a butter‐flavored diketone produced as a by‐product of yeast valine metabolism during beverage fermentation and affects evaluations (Krogerus & Gibson, 2013; Sato et al., 1981). Since the cognitive threshold of the diacetyl concentration in sake was reported to be 0.2 mg/L (Utsunomiya et al., 2004), its concentrations in the sake samples in Figure 4 indicate that sake prepared with only strain LP‐2 had a favorable value below the limit in the sensory score; on the other hand, those with strain LS‐4 exceeded the limit and were found to be unsuitable for *Yamahai‐shikomi* sake preparation. Diacetyl also has a typical unfavorable flavor in sake, beverages, and foods, while thought to be produced by LAB (Clark & Winter, 2015; Inoue, 2004). The general pathway for diacetyl synthesis is recognized to be a result of valine anabolism in bacteria and yeasts, since they arise from the spontaneous nonenzymatic oxidative decarboxylation of α‐acetolactic acid that is an intermediate in the valine biosynthesis pathway (Chuang & Collins, 1968; Horie et al., 2010; Suomalainen & Ronkainen, 1968). However, in the present study for *Yamahai‐shikomi* sake brewing, it was presumably produced by sake yeasts *S. cerevisiae* because most LAB had died off after completing the preparation of *Yamahai‐shubo* (Table 1). Although the detailed mechanism remains unknown, we assume that the coexistence of sake yeast with lactic acid bacteria may have changed its properties.

The significance of LAB as well as yeasts has been recognized in winemaking. In particular, the contribution of *Ll. plantarum* in malolactic fermentation is highly evaluated (Hernández et al., 2007; Mónica et al., 1999; Olsen et al., 1991). Recently, several reports have been published demonstrating that LAB in *Kimoto* (the same brewing method as *Yamahai‐shubo*) and *Sokujo‐shubo* affect sake qualities; e.g., yeasts grown in *Yamahai‐shubo* showed more enhanced tolerance to ethanol than those in *Sokujo‐shubo* (Iemura, Takahashi, et al., 1999; Iemura, Yamada, et al., 1999b; Taniguchi et al., 2020). LAB coexisting in Kimoto were found to gain more linoleic acid, which led to the higher enrichment of fatty acid content, including unsaturated double bonds in the phospholipid composition of yeast cell membranes, resulting in the acquisition of increased ethanol tolerance by the yeasts (Mizoguchi & Hara, 2010). Furthermore, bacteria found in arrested wine fermentations had a particularly strong prion‐like element (designated [*GAR* ^*+*^]) induction capacity (Jarosz et al., 2014), and it was presumed that something similar would be observed in sake brewing. In fact, LAB in *Kimoto* promoted the formation of [*GAR* ^+^] in yeast cells during the brewing of sake and might be beneficial in making *Yamahai‐shubo* sake (Watanabe et al., 2018). In addition, Takahashi et al. reported that the growth rate of *Ll. sakei* was influenced by the strain, pH, and temperature in *kimoto* preparation (Takahashi et al., 2021). It is assumed that there are various strains of *Ll. sakei* that grow predominantly in the *Yamahai‐shubo* or *Kimoto*. These reports demonstrate that LAB in *Yamahai‐shubo* significantly affect the properties of sake yeast as well as sake quality. The present study suggested that strain LP‐2 in *Yamahai‐shubo* has a more favorable effect on yeasts and *Yamahai‐shikomi* sake quality with strains 61‐02 and LM‐1. The mechanisms are being elucidated, adding more characteristics of LAB strains. We are trying to clarify the mechanism by which the characteristics of yeast change depending on the LAB coexisting in the *Yamahai‐shubo* so that we can improve the quality of *Yamahai‐shikomi* sake. Then, we would like to contribute to the diversification of sake quality of *Yamahai‐shikomi* sake by the differences in coexisting LAB (especially bacilli‐LAB).

## 5 CONCLUSION

We found that the addition of the nitrate‐reducing bacterium strain 61‐02, the cocci‐LAB strain LM‐1, and the bacilli‐LAB strain LP‐2 to *Yamahai‐shubo* produced *Yamahai‐shikomi* sake with more favorable aromas and tastes than that of another bacilli‐LAB strain, LS‐4. Furthermore, it is likely that strain LP‐2 in *Yamahai‐shubo* played a significant role in improving sake quality by reducing diacetyl more than strain LS‐4 in *Yamahai‐shikomi* sake brewing.

## FUNDING INFORMATION

There is no funding to report for this submission.

## CONFLICT OF INTEREST STATEMENT

The authors declare no conflict of interest associated with this manuscript.

## Data Availability

All data are incorporated into the article and its online supplementary material.
